# Pathogenic Mechanisms of Vaccine-Induced Immune Thrombotic Thrombocytopenia in People Receiving Anti-COVID-19 Adenoviral-Based Vaccines: A Proposal

**DOI:** 10.3389/fimmu.2021.728513

**Published:** 2021-08-13

**Authors:** Bruno Azzarone, Irene Veneziani, Lorenzo Moretta, Enrico Maggi

**Affiliations:** Immunology Research Area, IRCCS Bambino Gesù Children’s Hospital, IRCCS, Rome, Italy

**Keywords:** VITT, IL-6, PF-4, adenoviral vectors, erytrocyte, platelet, endothelia, mast cell

## Abstract

VITT is a rare, life-threatening syndrome characterized by thrombotic symptoms in combination with thrombocytopenia, which may occur in individuals receiving the first administration of adenoviral non replicating vectors (AVV) anti Covid19 vaccines. Vaccine-induced immune thrombotic thrombocytopenia (VITT) is characterized by high levels of serum IgG that bind PF4/polyanion complexes, thus triggering platelet activation. Therefore, identification of the fine pathophysiological mechanism by which vaccine components trigger platelet activation is mandatory. Herein, we propose a multistep mechanism involving both the AVV and the neo-synthetized Spike protein. The former can: i) spread rapidly into blood stream, ii), promote the early production of high levels of IL-6, iii) interact with erythrocytes, platelets, mast cells and endothelia, iv) favor the presence of extracellular DNA at the site of injection, v) activate platelets and mast cells to release PF4 and heparin. Moreover, AVV infection of mast cells may trigger aberrant inflammatory and immune responses in people affected by the mast cell activation syndrome (MCAS). The pre-existence of natural antibodies binding PF4/heparin complexes may amplify platelet activation and thrombotic events. Finally, neosynthesized Covid 19 Spike protein interacting with its ACE2 receptor on endothelia, platelets and leucocyte may trigger further thrombotic events unleashing the WITT syndrome.

## Introduction

Coronavirus disease 2019 (COVID-19) has been associated with more than four million deaths documented worldwide, thus vaccination against SARS-CoV-2 is the most relevant resource against the COVID-19 pandemic (1, 2).

At present, the European Medicines Agency (EMA) has approved two vaccines based on the messenger RNA (mRNA) technology: Pfizer BioNTech (BNT162b2) and Moderna COVID-19 (mRNA-1273). EMA has also approved two adenoviral vector (AVV)-based vaccines encoding the spike glycoprotein of SARS-CoV-2, namely COVID-19 Vaccine Janssen employing recombinant human adenovirus type 26 vector (Ad26.COV2.S b) and COVID-19 Vaccine AstraZeneca (Vaxzevria) employing the recombinant chimpanzee adenoviral [ChAdOx1-S] type 5 vector (3). At the beginning of April 2021, more than 82 million people in the European Union have been vaccinated and, among them, 20 million people have received Vaxzevria and Janssen vaccines (4). In addition, an undetermined number of Europeans received a vaccine other than the four EMA-approved vaccines, namely the Russian Sputnik employing the two different human Adenoviral vectors (type 5 and type 26). In this context, the chimpanzee adenoviral vectors have the desirable vector characteristics of human adenoviral vectors, but with negligible seroprevalence in the human population. Indeed, their employ in several Phase I clinical trials have shown good safety and immunological profiles. However, certain Chimpanzee AVVs may not be suitable for some populations since neutralizing antibodies to some chimpanzee adenovirus serotypes have been detected in humans from sub-Saharan Africa, Brazil and China (5).

With such a widespread diffusion of anti-COVID-19 vaccines, the detection of severe side effects associated with vaccine administration should be rapidly documented. In this context, from late February 2021, following the first report by Greinacher and coll., different groups highlighted several cases of an unusual syndrome termed vaccine-induced immune thrombotic thrombocytopenia (VITT) after vaccination with ChAdOx1 nCov-19 and Janssen vaccine but not with RNA vaccines (6–9), with a single exception. Indeed, a recent paper describes a VITT case after vaccination with mRNA Moderna vaccine that seems somehow atypical, since the patient was a 65 year old man, who developed the VITT syndrome after the second vaccine injection (10).

The clinical picture of moderate-to-severe thrombocytopenia and thrombotic complications at unusual sites was observed in healthy individuals under 60 years of age within 5 to 20 days after the first ChAdOx1 nCov-19 vaccination with a rate of 6.5 events per million (9). These clinical features characterize a syndrome that resembles the severe heparin-induced thrombocytopenia, a well-known pro-thrombotic disorder caused by platelet-activating antibodies that recognize a neo-antigen in the multi-molecular complexes formed by the cationic Platelet Factor 4 (PF4) and anionic heparin. The patients who develop VITT are characterized by high levels of IgG antibodies against PF4-polyanion complexes, despite they have never been exposed to heparin (4, 6–9).

Even if the risk of developing VITT symptoms does not appear to be higher than the basal risks in the general population, the mortality caused by cerebral venous thrombosis in patients who received Jannsen or Vaxzevria vaccine is higher than expected (8), with a rate of 0.37 events per million. Identification of the fine pathophysiological mechanism by which vaccine components trigger this rare syndrome is mandatory.

Firstly, it is important to understand why these thrombotic events occur at unusual sites. McGonagle and coll. propose a convincing explanation that cerebral sinus- and splanchnic veins, which drain the nasal sinus and intestines respectively, allow microbiota and viral products to enter the endothelial networks of lining vessels. In this context, the undue presence in these sites of high titers of anti-PF4 pathogenic autoantibodies may lead to an aberrant immune response including activation of platelets, mast cells and neutrophils, increased platelet consumption and thrombosis (11).

Greinacher and coll. ask a series of basic questions wondering whether these anti-PF4 antibodies are autoantibodies induced in bystander autoreactive B cells by the strong inflammatory stimulus of vaccination, or antibodies induced by the vaccine cross-reacting with PF4 and platelets (4). However, since in healthy donors PF4 is hidden in the α granules of platelets (12), an early event leading to a specific anti-PF4 antibody response would be necessarily a rapid platelet activation with subsequent release of PF4 outside the platelets soon after the vaccination. Thus, in our opinion, the preliminary basic question concerns the mechanism causing platelets activation soon after vaccine administration.

Regarding this point, Greinacher and coll. speculate that interactions between the vaccine components and platelets could play a role in the pathogenesis (4). However, they exclude a role of the adenoviral vector, arguing that the amount of adenovirus in a 500-microliter vaccine injection administered 1-3 weeks earlier could hardly contribute to subsequent platelet activation observed in these patients (4).

They reiterate this statement although they show enhanced reactivity of patients’ sera with platelets in the presence of ChAdOx1 nCov-19 and quote references showing that adenovirus binds to platelet and causes platelet activation (4). They rather consider that a possible trigger of these PF4-reactive antibodies could be free DNA present in the vaccine, since they had previously shown that DNA and RNA can form multi-molecular complexes with PF4, which binds antibodies from patients with heparin-induced thrombocytopenia (4). Once more, the neglected point is: which event causes platelet activation and PF4 exposure?

Herein, we discuss about a possible role of the adenoviral vector in platelet activation, analyzing what was previously published on this matter and suggesting some relationship with the induction of VITT.

## Preclinical Models

Several studies using murine and rabbit models have shown that soon after intravenous injection of adenoviral vectors the main target in the blood is represented by platelets as they express the Cocksackie/Adenovirus receptor (CAR). Adenoviral particles engulfed by platelets mediate platelet activation, as revealed by the surface exposure of the adhesion molecule p-Selectin that, interacting with its ligand PSGL-1 on leukocytes, favors platelet-leukocyte aggregate formation. At the same time, adenoviruses induce endothelial cell activation, as shown by VCAM-1 expression on virus-treated cultured endothelial cells and by the release of ultra-large molecular weight multimers of Von Willebrand Factor (VWF) within 1-2 hours from virus addition to cultures (13–16). Moreover, CAR expressed on endothelial cells acts as a mechanic sensor responsive to fluid shear stress, activating endothelial mechanical transduction, modulating endothelial function leading to vascular pathophysiologic events (17). Accordingly, it is conceivable that adenoviral vectors injected intravenously may rapidly activate both platelets and endothelia and trigger a complex platelet-leukocyte-endothelial axis resulting in coagulation abnormalities and severe thrombocytopenia from 5 to 24 hours after adenovirus delivery, in a vector dose-dependent manner (13–17). Similar effects were observed injecting AVVs to non-human primates (18–20). Indeed, this treatment causes a rapid temporal and dose-dependent thrombocytopenia, associated to the reduction of erythrocytes counts, since erythrocytes could bind activated platelets and be cleared and/or sequestered along with them. In addition, these animals show dose-dependent elongation of clotting times with concomitant increase of fibrinogen and of the amount and size of VWF multimers, which, likely, reflect endothelial cell damage (18–20).

## Human Trials Based on Adenoviral Vectors

In humans, natural adenovirus infections have not been associated to altered coagulation, even if a viremic phase has been described, with the exception of rare severe cases of detectable high levels of the virus (21–24). Some human adenovirus serotypes persist in lymphoid tissues for several years, through a form of low-grade replication, and the Group C adenovirus DNA has been identified in peripheral blood lymphocytes during fatal acute infection and in immunosuppressed patients (25, 26). Moreover, *in vitro* experiments performed with blood from healthy donors show that the main target of the adenoviral vectors is human erythrocytes and, at a much lesser extent, platelets (27). This occurs since, differently from murine and monkey red blood cells, human erythrocytes express CAR and Complement Receptor 1 (CR1), which mediate their binding to adenoviral vectors (28, 29). In another study, despite a variable pattern of distribution between donors, the relative amount of virus associated with platelets *in vitro* was significantly higher than that recovered in erythrocytes (30). This difference is possibly due to the different type of anti-coagulant drugs employed in blood sampling (29, 30).

In neoplastic patients treated with intra-tumor infusions of adenoviral vectors, the number of viral genome copy number generally peaks between 9 to 12 hours after infusion and is followed by viral shedding into the blood stream within 24 hours. By comparing serum and blood clots, it was showed that in these patients viral DNA detection was higher in blood clots. Analysis of interactions between oncolytic adenoviruses and blood cells was performed *in vivo* and viruses resulted mainly associated with erythrocytes or granulocytes, but poorly associated with platelets. Nevertheless, in cancer patients, the absence or low amount of virus in the platelet compartment 24 hours after treatment was associated with decreased thrombocyte and leukocyte counts (29).

Even though these results could apparently recall data of preclinical models, we propose an alternative mechanism. It has been shown that damaged erythrocytes can modulate platelet* *reactivity directly through either chemical signaling or adhesive erythrocyte*-*platelet interactions, contributing to high risk of thrombotic complications (31). Adenovirus may bind vitamin K-dependent coagulation factors and several complement (C’) components. For instance, AVV can induce the alternative C’ pathway by binding C3 directly, subsequently C3–AVV complexes can be delivered to CR1 clustered on the surface of human erythrocytes (24). In addition, some adenovirus types binding CAR induce hemagglutination of human erythrocytes (32). Thus, we propose that the above mentioned adenovirus-erythrocytes complexes, which may per se trigger the clotting pathway (24), could also indirectly or directly activate platelets that (24, 29, 31), in turn, could form aggregates with leukocytes leading to subsequent clearance from the blood stream and blood clots formation.

Results of a phase I clinical trial with AVV administered into the right hepatic artery of subjects with a partial deficiency of ornithine trans-carbamylase (OTCD) show that patients exhibited reversible thrombocytopenia and anemia. Viral blood dissemination was detected during vector infusion and was still detectable eight hours after infusion but not later (30).

## A Multistep Process Involving the Adenoviral Vector May Lead to VITT

Overall, these data indicate that adenoviral vectors may cause thrombocytopenia and coagulopathies both in pre-clinical models and in human individuals, even though the blood cell targets are not the same.

Therefore, it is possible that they will play some role in the cause of the rare VITT syndrome in some individuals after the first administration of anti-COVID-19 vaccines employing such vectors. However, in agreement with the hypothesis of Greinacher and coll. (4), some additional variables should be considered: i. the vaccine components, ii. the injection site and bio-distribution of vectors, iii. the timing of symptoms’ onset, iv. the pre-existence of natural antibodies recognizing the PF4/heparin complexes, v. the role of the neo-synthetized Spike protein.

## Vaccine Components

The ethylene diamino tetra acetic acid (EDTA) enters in the composition of the Vaxzevria vaccine buffer (33) but not of Ad26.COV2.S vaccine (9), and it may cause both platelet activation and capillary permeability (34, 35). However, it seems highly unlikely that the amount of EDTA present in a 500-microliter vaccine injection administered intra-muscularly may cause extended damages to platelets circulating in the blood stream and distant endothelia.

The chimpanzee adenoviral [ChAdOx1-S] vectors are cultured and expanded by using the immortalized embryonic kidney cell line - REx HEK293 cells. Even though the product is nuclease-treated and further purified (33), some vaccine preparations could still contain very few residual cellular proteins and nucleic acids that exceptionally could exert platelet activation.

The AVV itself has the potential to activate platelets and to damage vessels; therefore, its direct involvement cannot be excluded, differently from what has been proposed (4).

## The Site of Injection and Biodistribution of Vectors

Most vaccines are given *via* the intramuscular route into the deltoid or the anterolateral part of the thigh. This optimizes the immunogenicity of the vaccine and minimizes adverse reactions at the injection site (36). However, we have limited information on the fate of the vaccine during 48 h post-intramuscular injection (24). In addition, the muscle environment conditions the type of cells that are recruited, their cytokines secretion, and how such recruited cells and extracellular proteins impact on inflammatory and the immune responses. For instance, occasional local micro trauma and micro bleeding can favor the appearance of negatively charged extracellular DNA, which may act as a powerful adjuvant conditioning the local immune response (11). Nevertheless, muscle, because of its abundant blood supply, favors *the correct* delivery into the circulation optimizing mobilization and processing of viral vectors (36).

Thus, the AVV of Vaxzevria and COVID-19 Vaccine Janssen are supposed to efficiently reach the blood stream after intramuscular injection. In this context, pharmacokinetics studies on AVV titers and persistence in the blood and inside the blood cells become mandatory, in order to determine the extent of their interactions with erythrocytes and platelets.

## Timing of the Symptoms Onset

Initial symptoms of VITT may occur as early as 5 up to 24 days after vaccination, while IgM to IgG switch does not occur in such a short interval after the first vaccination. Thus, we postulate that these patients have pre-existing B cells producing antibodies recognizing the PF4/heparin complexes. This assumption is based on recently published results showing the frequent detection in healthy donors of B cells producing germline natural anti-PF4/heparin antibodies primarily of IgM isotype, detectable in cord and adult peripheral blood. These antibodies are part of the natural IgM germline repertoire and may be synthesized even without previous antigen contact. These B cells could respond to non-specific stimuli leading to production of IgM reacting with PF4/heparin complexes. In addition, natural IgG antibodies able to bind PF4/heparin complexes can be detected in sera of the 4.3-6.6% of general population usually associated with the presence of chronic bacterial infections, such as periodontal disease (37, 38). Importantly, a subset of these antibodies with high affinity, clustering PF4-molecules, can form neo-antigenic complexes recognized by polyanion-dependent anti-PF4/P-antibodies in the absence of heparin. Most individuals with these anti-PF4/polyanion antibodies remain asymptomatic, likely because their titers are too low to have clinical significance or because they are non-pathogenic “mimicking” antibodies, which are well-known to be frequently produced by the immune system (39, 40). However, in very rare vaccinated individuals, such natural IgM or IgG antibodies, if present at a clinically relevant titer, could bind PF4 or PF4 complexes formed after the release of PF4 from adenovirus-infected platelets. Alternatively, the AVV present in the blood stream may interact with red blood cells (27) generating altered erythrocytes (29, 31) that, in turn, are able to activate platelets (31), thus starting a vicious circle.

If the symptoms occur between 10 and 24 days it is conceivable that vaccinated people do not possess natural anti-PF4 antibodies, since, during this period, the immune system may mount a T cell-dependent anti-PF4 antibody response with IgM to IgG isotype switching.

The pathological reaction leading to VITT would be similar in both situations.

We suggest that, essentially in 20% of individuals suffering from immediate strong local inflammatory reaction (41), an early event may be represented by a robust production of the inflammatory cytokine interleukin 6 (IL-6), which has been shown to be increased in all patients receiving AVVs for the treatment of the OTCD genetic syndrome (30). In COVID-19 patients, high levels of IL-6 may favor the hyper inflammatory reaction with further damage of endothelia, likely leading to the final multi-organ dysfunction associated with severely dysregulated coagulation  (42).

Another early event could be represented by the adenoviral dissemination into the blood stream few hours after injection (30). AVV, binding primarily erythrocytes, mast cells and, at a lesser extent, platelets and endothelia could favor a cascade of interactions that, in rare instances, could evolve up to severe thrombotic events.

Erythrocytes may be damaged by the AVV (27–29, 31), favoring platelet activation and their interaction with endothelia also damaged by IL-6 and AVV themselves. Activated platelets degranulate, delivering into the blood stream the content of their α-granules, mainly PF4, that could form complexes with natural heparin released by basophils and mast-cells upon activation by IL-33 produced by damaged endothelia (43).

In this context, mast cells may be far more integrally involved in the development of COVID-19 VITT, since they express the adenoviral receptor (CAR) and AVV infect mast cells with a great efficiency (44). Moreover, the mast cell activation syndrome (MCAS) (45), is characterized by somatic mutations in multiple mast cell regulatory genes driving chronic aberrant constitutive and reactive mast cell activation, with excessive inflammatory reactions and aberrant heparin release (45). Moreover, MCAS in few instances drives the development of pathogenic autoantibodies triggering true autoimmune diseases (46).

Thus, MCAS patients, reacting aberrantly with the vector *via* its spurious effects on the humoral immune system, may produce pathogenic anti-PF4/heparin antibodies; this represents a key factor able to explain the rarity of COVID-19 VITT.

Additionally, positively charged circulating PF4 may interact with negatively charged extracellular DNA at the site of vaccine injection, forming positively charged DNA-PF4 complexes which, taken up by antigen presenting cells, will stimulate strong interferon response *via* Toll-Like Receptor 9. Subsequently, memory B cell engagement in the regional lymph-nodes, will cause increased PF4 autoantibody production, that, upon repeated interaction with PF4, will trigger extensive FcRγII mediated platelet activation, initiating a vicious circle leading to severe thrombotic events (11). At present, the epidemiology of MCAS is quite preliminary and its prevalence has been estimated by various authors (45, 47) ranging from rare (00.1%) to substantially prevalent (**~**17%).

A further step may be the presence of natural or T-cell dependent IgG antibodies in 4.3-6.6% of general population (37, 38) that bind PF4 or PF4/heparin complexes with subsequent platelet activation. It has been reported that if the percentage of activated platelets remains below 7.6% threshold, no events mimicking the pro-thrombotic adverse drug reaction similar to heparin-induced thrombocytopenia (HIT) would develop. In contrast, if platelet activation is higher than 7.6% threshold, a possible evolution to HIT may occur (48). Combining the frequency of individuals exhibiting anti-PF4/heparin complexes (37, 38) with the frequency of individuals presenting the MCAS (45) the probability of individuals presenting both alterations would range 1 in 200.000 to 400.000 subjects, approaching to the frequency of vaccinated individuals who may develop thrombotic complications. If we further restrict this possibility to the 20% of vaccinated subjects exhibiting a strong local inflammation (41), we will find a theoretic frequency ranging between 1 in 1x10^6^ to 2x10^6^ subjects, which roughly corresponds to the percentage of patients developing VITT (8, 9).

Finally, the situation could have worsened by the neo-synthesis of the Spike protein (allowing an efficient vaccination). SARS-CoV-2 binds through its Spike protein to the angiotensin-converting enzyme-2 (ACE2), which is expressed by monocytes/macrophages, mast cells, epithelial and endothelial cells of different organs (48). In endothelial cells, Spike/ACE2 interaction impairs the activity of ACE2. This causes the indirect activation of the kallikrein–bradykinin pathway, with subsequent altered endothelial leakage and vascular permeability (49). This event triggers a number of pro-inflammatory mechanisms further increasing vascular damage and permeability with the potential development of disseminated intravascular coagulation (DIC). Moreover, platelets express high levels of ACE2 and, as a consequence, purified Spike protein may directly activate platelets, potentiating the prothrombotic cascade (9). Finally, since MCAS may be intimately involved in the pathogenesis of both severe form of acute COVID-19 infection and of COVID-19 “long-haul” syndrome through ACE2/Spike protein interactions (50, 51), it may be speculated that the interplay of altered mast cells with neosynthesized SARS-CoV-2 spike protein could further contribute to VITT development.

In vaccinated patients, neo-synthesized Spike proteins should be efficiently processed and degraded by antigen-presenting cells. However, it cannot be excluded that some neo-synthesized Spike proteins may escape the immune machinery and be released under free form in the blood stream.

**Figure 1:**
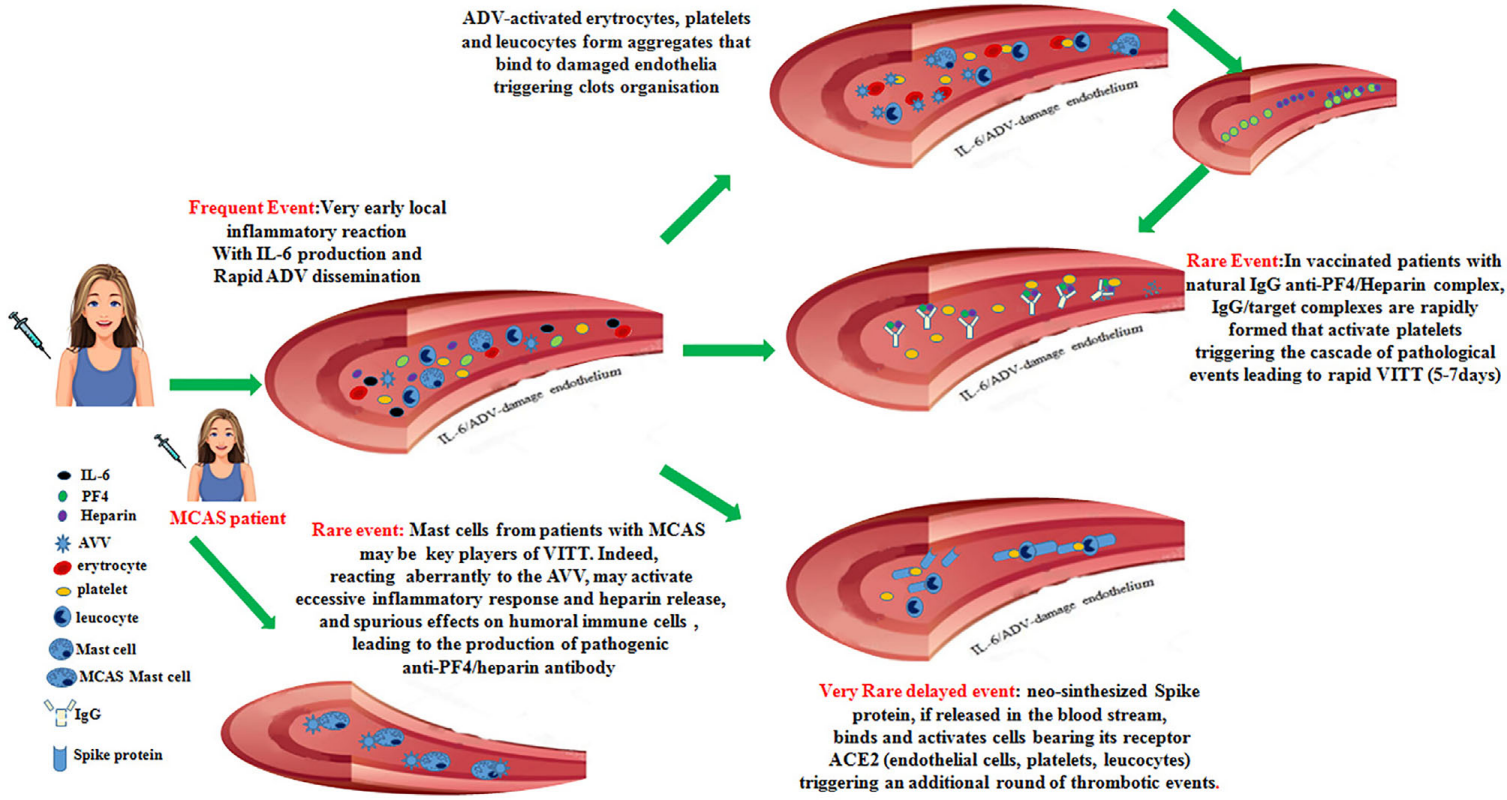
Recapitulates the cascade of pathogenic events that could favor the onset of the vaccine-induced immune thrombotic thrombocytopenia in individuals vaccinated with anti-COVID-19 adenoviral-based vaccines.

Thus, in some vaccinated individuals with a predisposing environment, neo-synthesized Spike protein acting on inflammatory cells, platelets and damaged endothelia could yield an additional trigger critical to unleash a delayed onset VITT syndrome. The set of these events is summarized in **Figure 1**.

## Discussion

In this contribution, based on confirmed data from literature, we propose a cascade of events that, in very few individuals receiving the first administration of anti-COVID19 vaccines based on AVV, could favor the onset of different degrees of coagulopathies up to the development of the VITT syndrome. Possible checkpoints that could hinder this multistep process are represented by: i. the presence of natural antibodies able to bind anti PF4/heparin complexes, ii. the levels of IL-6 produced in the first hours after vaccination, iii. the extent and the persistence of the adenoviral vectors into the blood stream, iv. the percentage of activated platelets, v. the presence of subjects presenting MCAS, vi. the amount of neo-synthesized Spike protein interacting with receptors on endothelial cells. If only some of these events occur this might lead to the development of intermediate thrombotic events, while, if all of them occur, the VITT syndrome might develop. Of note, most of vaccinated individuals undergoing thrombotic events up to VITT are young fertile women. In these patients, some events could favor the development of coagulopathies: i. the large use of contraceptive estrogens, which may facilitate thrombotic events, ii. the recent surgery, which may lead to spontaneous PF4/heparin antibodies production in the absence of exogenous heparin (38): it is also questionable if pregnancy may favor this condition, iii. the autoimmune disorders associated with anti-phospholipids and anti-platelets autoantibodies production are much more frequent in women.

This review does not absolutely intend to criticize adenoviral-based vaccines, which revealed of primary importance in the COVID19 pandemic. Our goal was to try to clarify the pathogenic mechanisms leading to VITT with the perspective of improving the handling of the vaccine-recipients.

## Data Availability Statement

The original contributions presented in the study are included in the article/supplementary material. Further inquiries can be directed to the corresponding authors.

## Author Contributions

BA, LM, and EM imagined, planified and wrote this Perspective Article. IV imagined and composed the figure. All authors contributed to the article and approved the submitted version.

## Funding

This work was supported by grants from AIRC Special Program Metastatic disease: the key unmet need in oncology 5 per mille 2018 ID 21147 to Lorenzo Moretta.

## Conflict of Interest

The authors declare that the research was conducted in the absence of any commercial or financial relationships that could be construed as a potential conflict of interest.

## Publisher’s Note

All claims expressed in this article are solely those of the authors and do not necessarily represent those of their affiliated organizations, or those of the publisher, the editors and the reviewers. Any product that may be evaluated in this article, or claim that may be made by its manufacturer, is not guaranteed or endorsed by the publisher.
